# Cellulose polymers with β-amino ester pendant group: design, synthesis, molecular docking and application in adsorption of toxic metals from wastewater

**DOI:** 10.1186/s13065-022-00837-7

**Published:** 2022-06-10

**Authors:** Noor Nairat, Othman Hamed, Avni Berisha, Shehdeh Jodeh, Manuel Algarra, Khalil Azzaoui, Omar Dagdag, Subhi Samhan

**Affiliations:** 1 Chemistry Department, Faculty of Science, An-Najah National University, P.O. Box 7, Nablus, Palestine; 2 Department of Chemistry, Faculty of Natural and Mathematics Science, University of Prishtina, Prishtina, 10000 Kosovo; 3 Materials Science-Nanochemistry Research Group, NanoAlb-Unit of Albanian Nanoscience and Nanotechnology, 1000 Tirana, Albania; 4 Department of Science, INAMAT2 Institute for Advanced Materials and Mathematics, Public University of Navarra, Campus of Arrosadia, 31006 Pamplona, Spain; 5 Laboratory of Mineral Solid and Analytical Chemistry LMSAC, Department of Chemistry, Faculty of Sciences, Mohamed 1st University, P.O. Box 717, Oujda, Morocco; 6 Institute of Nanotechnology and Water Sustainability, College of Science, Engineering and Technology, University of South Africa, Johannesburg, South Africa; 7 Palestine Water Authority, Ramallah, 00001 Palestine

**Keywords:** Water treatment, Persistent pesticides, Difenoconazole, Cellulose nanocrystalline, 2-furan carbonyl chloride, Cellulose, Monte Carlo, Glycine, Molecular dynamic, Adsorption, Wastewater

## Abstract

**Background:**

Cellulose polymers with multidentate chelating functionalities that have high efficiency for toxic metal ions present in water were designed, synthesized, and analyzed. The synthesis was carried out by reacting microcrystalline cellulose extracted from the solid waste of the olive industry with tert-Butyl acetoacetate (Cell-AA), produced cellulose with β-ketoester functionality was then reacted with aniline and the amino acid glycine to produce Cell-β-AN and Cell-β-GL, respectively.

**Results:**

The adsorption efficiency of the three polymers toward Pb(II) and various toxic metal ions present in sewage was evaluated as a function of adsorbent dose, time, temperature, pH value, and initial ion concentration to determine optimum adsorption conditions. The three polymers showed excellent efficiency toward about 20 metal ions present in a sewage sample collected from the sewer. The adsorption process follows the Langmuir adsorption isotherm model with a second-order of adsorption rate, the calculated qe values (2.675, 15.252, 20.856 mg/g) were close to the experimental qe values (2.133, 13.91, 18.786 mg/g) for the three polymers Cell-AA, Cell-β-AG and Cell-β-AN, respectively. Molecular Dynamic (MD) and Monte Carlo (MC) simulations were performed on the three polymers complexed with Pb(II).

**Conclusion:**

The waste material of the olive industry was used as a precursor for making the target cellulose polymers with β-Amino Ester Pendant Group. The polymer was characterized by SEM, proton NMR, TGA, and FT-IR spectroscopy. The efficacy of adsorption was quantitative for metal ions present in a real sample of wastewater and the efficiency didn’t drop even after 7 cycles of use. The results indicate the existence of strong complexation. The thermodynamic study results showed a spontaneous bonding between of Pb(II) and the polymers pendant groups expressed by the negative value of the Gibbs free energy.

## Introduction

Water contamination has become a critical global problem and a major health issue for living organisms and ecosystems. The issue was related to [1–3] industrial waste, agricultural waste, and the household cleaning items that release toxic heavy metals, organic material, dyes and other to the sewage system [4–6]. Other sources of toxic contaminants include medical, agricultural, plumbing, body care products. Among the toxic heavy metal ions that pose a risk and required immediate attention are Cd(II), Cu(II), Mn(II), Mg(II)_,_ Sr(II), Al(II), Co(II), Ni(II), Cr(III), Zn(II) and Pb(II) ions [7, 8]. Recycling of wastewater released from industrial and human activities has become a necessity. Among the most effective technologies used in wastewater purification and recycling wastewater from toxic heavy metals and other contaminants are precipitation, membrane filtration, electrodialysis, electrochemical treatment, coagulation [9], flotation [10], cementation [11], solvent-solvent extraction [12], ion exchange [13], chemical oxidation [14], reverse osmosis [15], and adsorption [16]. Amon these methods, adsorption received the highest attention due to its simplicity, availability, profitability, practicality, ecofriendly, recyclability, relatively low cost, less sludge production, high efficiency, and high selectivity [17–19]. The adsorption method mainly focuses on activated carbon as the adsorbents. However, some withdraw backs such as processing costs have led the scientist to search for other means [20]. More desirable adsorbents are those made from renewable, low-cost materials, especially those derived from agricultural waste materials [21] and biological adsorbent [22, 23]. Natural based adsorbents made from cellulose [24], lignin, chitosan and hemicellulose received the most attention. They are economically feasible, environmentally friendly, and highly efficient for removal of heavy metal ions from wastewater [25]. Cellulosic based adsorbents and related obtained from waste materials such as Kenaf [26, 27], cotton linters [28], wheat straw [29], wood sawdust [30], rice husk [31] were prepared and investigated for the adsorption of several metals such as those mentioned above. Nanoparticle adsorbents made from natural materials such as cellulose nanocrystalline (CNC) were the most promising, especially. Despite all the rapid progress in the nanocellulose adsorbents still many celluloses-based materials and derivatives have not been explored in wastewater purification. In this work, new cellulose-based adsorbents were prepared and used in wastewater purification. The new adsorbents were prepared by functionalizing microcrystalline cellulose with β-ketoester to form cellulose with 1,3-dicarbonyl pendant group, which then converted to a Schiff base by reacting it with aniline and the amino acid glycine. Microcrystalline cellulose used in this work was extracted from olive industry solid waste (OISW) [32, 33]. The prepared polymer showed an excellent affinity for various heavy metals since the functional groups are considered multidentate chelating agents. The adsorption efficiency of the three cellulose-based polymers was evaluated toward Pb(II) and other metal ions from real wastewater samples.

## Experimental

### Material

All chemicals and reagents used in this work were purchased from Sigma-Aldrich chemical company (Jerusalem) and used as received. The chemicals include tert-Butyl acetoacetate (t-BAA), lithium chloride anhydrous (LiCl), N,N-dimethylacetamide anhydrous (DMAc), aniline, glycine, lead(II) nitrate, acetic acid, methanol and nitrogen gas (purity 99.9%). All reagents used were of analytical grade. Deionized water was used to prepare all solutions. Cellulose used in this work was extracted from olive industry solid waste (Jeft) by a chemical process that was developed at the laboratories of An-Najah National University-Nablus/Palestine.

### Methods

#### Characterization

Nicolet 6700 Fourier Transform Infrared (FT-IR) spectrometer equipped with the Smart Split Pea micro-ATR accessory (Thermo Fisher Scientific, Waltham, MA, USA) was used in this work. The following IR parameters were used: resolution 4 cm^-1^, spectral range 400–4000 cm^-1^, number of scans 128. Thermo-gravimetric analysis (TGA) and differential scanning calorimetry (DSC) measurements were performed using a TG/DSC Star System (Mettler-Toledo) coupled with a MS-Thermostar GSD320 (Pfeiffer Vacuum) Mass Spectrometer. TG/DSC analysis was performed with Pt crucibles, in N_2_ flow (20 mLmin^−1^) at a heating rate of 5 ºC min^−1^ in the range 25–1100 ºC by a HT1100 oven connected to a MX5 microbalance (thermostatic at 22 ºC). The STARE software v.10.0 (Mettler Toledo) controlled the process.

Metal ions concentrations were determined using Flame Atomic Absorption Spectrometer (FAAS, ICE3500 AA System, Thermo scientific, United Kingdom) and the inductively coupled plasma mass spectrometry (ICP-MS) *via* an iCAP^TM^ RQ ICP-MS (Thermo Fisher Scientific, Waltham, MA, USA). All analysis studies were performed in triplicate and the mean of the three runs was reported. The error range in the experimental data was analyzed using Excel Microsoft software, a certainty interval of 95% was used. The data analysis was performed using the t-test. All variations were considered statistically when p ˂ 0.05 for the analysis of t-test. The flame type was air-C_2_H_2_.

### Preparation of cellulose acetoacetate (Cell-AA)

A sample of microcrystalline cellulose (5.0 g, 0.15 mol/anhydrous glucose repeat unit) was added to a 0.5 L one necked round bottomed flask containing 200.0 ml distilled water and stirred magnetically for 2 h at room temperature. The cellulose was collected from water by suction filtration then suspended in 200 ml methanol for one hour. This process was repeated three times to activate the cellulose and remove water. The activated cellulose was collected by suction filtration then suspended in a 130.0 ml anhydrous DMAc two times, the first time was done for an hour, while the second time was carried out overnight. The activated cellulose was then collected by suction filtration and transferred to was added to a solution of LiCl in DMAc (8.0%, 150 mL) prepared by dissolving a 9.75 g of anhydrous LiCl in a 150 ml DMAc (2.697 mol) in a 500 ml round bottomed flask equipped with a magnetic stir bar and condenser, the flask was connected to a trap via the condenser and kept under nitrogen gas. The mixture was stirred at room temperature until a clear solution was obtained (about two hours). Then, a 33.5 ml of a t-butyl acetoacetate (t-BAA) (9.6 g, 61.5 mmol) was added dropwise to the solution under a blanket of nitrogen and heated to 120 ºC using oil bath in a 2 h period and stirred overnight. The reaction was transferred to a 1 L beaker, then 500 ml of distilled water was added dropwise to the reaction and then placed in the refrigerator overnight.

The resulted gel was filtered by suction filtration then transferred to a 1000 ml beaker containing 500 ml of methanol for washing. This step was repeated twice, the first stirring was done for 15 min, while the second one was carried out for 30 min. Product was collected by suction filtration and dried at 100 ºC, yield was about 86.7%.

### Preparation of cellulose β-aniline ester (Cell-β-AN)

A 2.0 g sample of cell-AA polymer was suspending in a 100 ml methanol, then 2.0 ml (2.04 g, 21.9 mmol) of aniline was added in one portion, followed with a 2.0 ml (2.1 g, 34.96 mmol) of acetic acid, added at once as a catalyst. The mixture was refluxed for 8 h. The resulting polymer was filtered, washed with water (3 x 20 mL) and dried in an oven at 110 ºC.

### Preparation of cellulose β-glycine ester (Cell-β-GL)

A 2.0 g of cell-AA polymer was suspended in acetic acid (40 ml), then 2.0 g (1.587 ml, 26.6 mmol) of glycine was added at once. Reflux was done for 6 h at 70 ºC. The resulting polymer was washed two times with water, diluted solution of sodium bicarbonate (1.0%), water and finally two times with methanol, and dried at 90 ºC.

### Adsorption study

All experiments were performed in plastic vials (50 ml each) that were held in a shaker and placed in a water bath equipped with a thermostat. The effect of various variable such as metal ion concentration (C_0_), adsorption time, pH value, adsorbent dosage, and temperature on adsorption efficiency was evaluated. The adsorption study was performed on the Pb(II) metal ion. A sample of each mixture was withdrawn using a 5.0 ml plastic syringe, then filtered through a 0.45 µm syringe filter. The collected filtrate was analyzed by FAAS at 217.0 nm for determining the residual metal ion concentration and thus to determine the adsorption efficiency according to Eq. 1 and 2, respectively.

1 $$ R\,\left( {\text{\% }} \right)\, = \,\frac{{C_{o} - C_{e} }}{{C_{o} }}100 $$

2 $$ Q_{e} = \frac{{C_{o} - C_{e} }}{m}V $$

C_0_ and C_e_ are the initial and equilibrium concentration in ppm of metal ion in solution respectively, Q_e_ (ppm) is the equilibrium adsorption capacity of the adsorbent (mg/g), m is the weight of the adsorbent (g), and V is the volume of the solution (L).

### Wastewater purification

A sample of sewage water collected from the Beit Dajan wastewater purification planet (Nablus-Palestine) was used in this study. The sample was first analyzed by ICP-AES (Water Center, An-Najah National University, Nablus, Palestine) to determine the metals content and their concentrations. Then three 10 ml samples of the wastewater were placed in two Erlenmeyer flasks, a 100 mg of each cellulose-based polymer (Cell-AA, Cell-β-AN, Cell-β-GL) was added to each flask. The pH of the solution was adjusted to 8.0. The mixtures were shaken at room temperature for 30 min using a thermostat shaker. A 5.0 mL sample of each mixture was withdrawn and filtered through a 0.45 µm syringe filter and analyzed by ICP-AES for residual metal ions concentrations.

### DFT calculations

DFT was performed using the Dmol3 software. Geometry optimization (spin unrestricted) using the double numerical plus polarization basis set (DNP) along with the PBE functional within the m-GGA approximation is used. Grimee DFT-D was used to provide dispersion correction effects. The COSMO method is used to include water as a solvent [34–37]. For the ELF—analysis, a single point geometry calculation (using geometry coordinates generated by the Dmol^3^ software in the previous step) was performed using the Orca software [38] at the density functional theory level with the M06 exchange-correlation functional and the def2-TZVP basis set [39]. The van der Waals interactions were accounted for by an atom-pair dispersion correction using the zero-damping scheme (D30) [40]. The adsorption energy is evaluated using the well-known method [41–44]. The non-covalent interaction (NCI) was calculated using Multiwfn software [45]. The NCI surface is plotted using software the Visual Molecular Dynamics [46].

Molecular Dynamic simulations were with the universal force field [47] to obtain detailed molecular details to elucidate the adsorption process of the between lead ions and the Cell-β-AN or Cell-β-GL surface. The adsorption is modelled using an 8 monomeric cellulose unit with a side chain modified by 8 GL or AN moiety, containing an upper layer composed of 400 water molecules and 3 Pb(II) ions. The MD is performed under NVT ensemble at 298.15 K, with 1 fs time step and a total simulation time of 2500 ps [37, 48–57]. A Nose thermostat is used for temperature control [58].

## Result and discussion

### Synthesis of cellulose-acetoacetate polymers (Cell-AA)

**Fig. 1:**
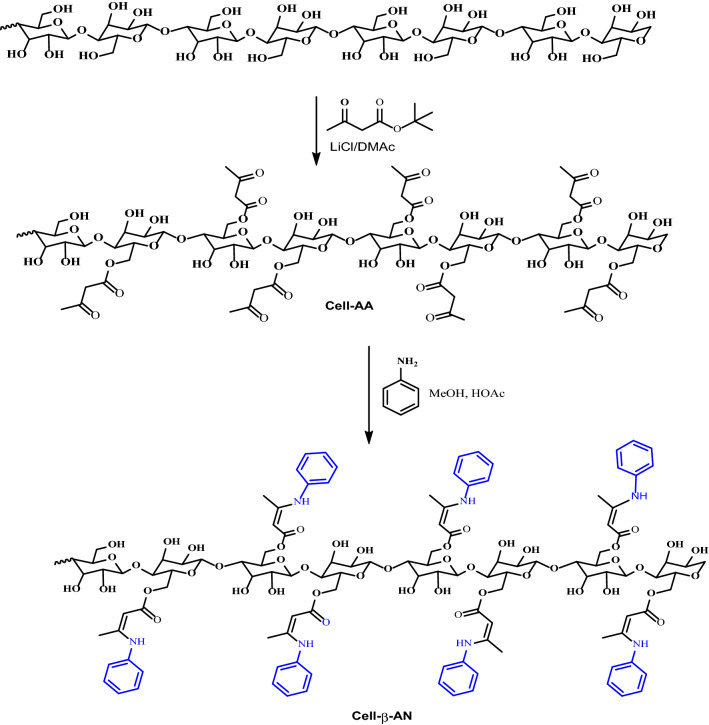
Preparation of cellulose acetoacetate from Cellulose and t-BAA

Cellulose functionalized with acetoacetate group was prepared according to a procedure reported in the literature with major modification [59]. Cellulose was dissolved in 8.0 wt% LiCl/DMAc solution at room temperature then reacted with tert-butyl β-ketoester at about 110 °C for 6 h (Fig. 1). The yield after product purification was about 86.7%.

**Fig. 2:**
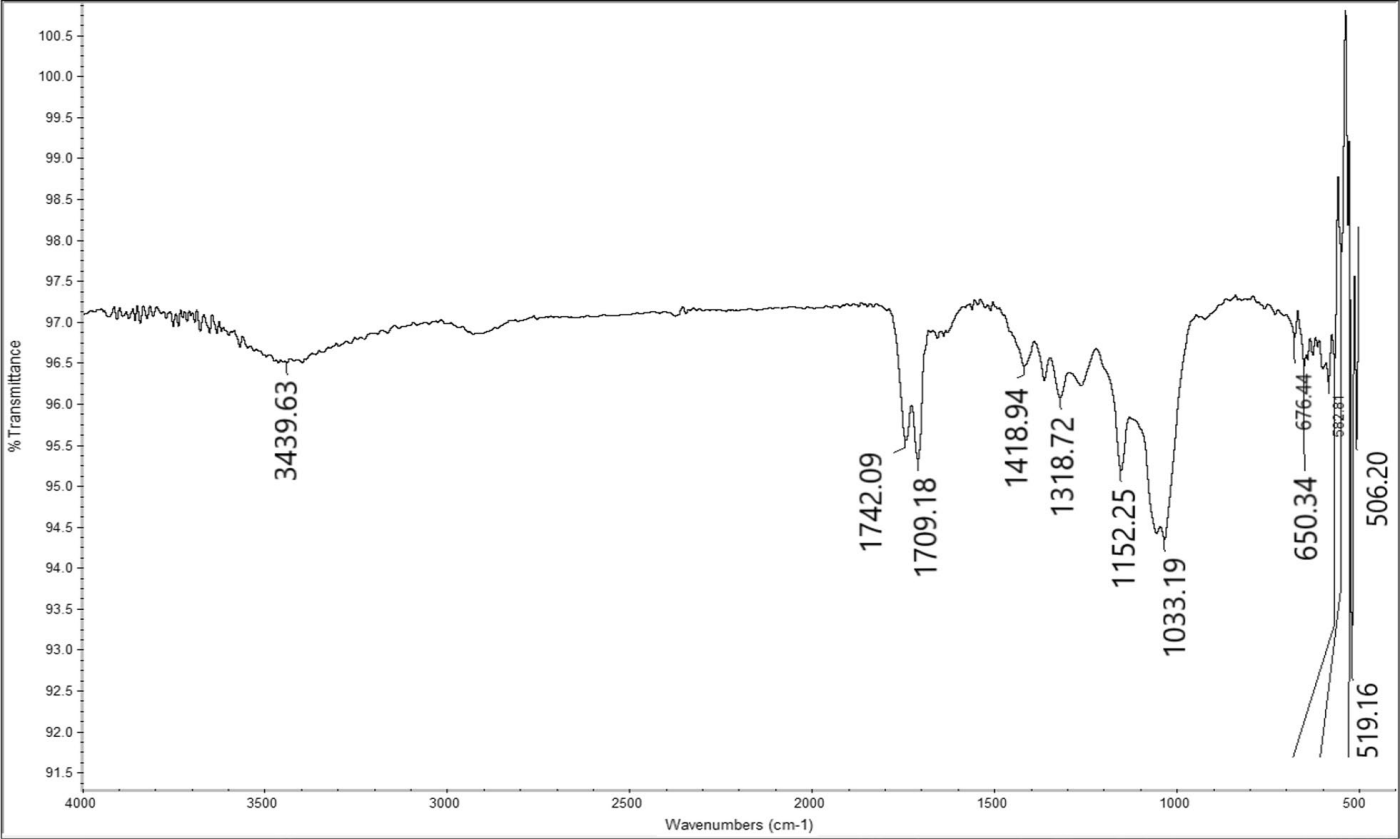
FT-IR spectrum for Cell-AA

The prepared Cell-AA was characterized by FT-IR, obtained spectrum is shown in Fig. 2. The most significant peaks for Cell-AA were observed at 1742 and 1709 cm^-1^ assigned to the carbonyls of ester and ketone, respectively. The IR spectrum also shows three bands at about 1152, 1057 and 1033 cm^-1^ corresponding to the vibration of C–O–C of ester, pyranose ring skeletal and to β-glycosidic linkage respectively. The broad peak at 3439 cm^-1^ attributed to the O-H bond stretching.

**Fig. 3:**
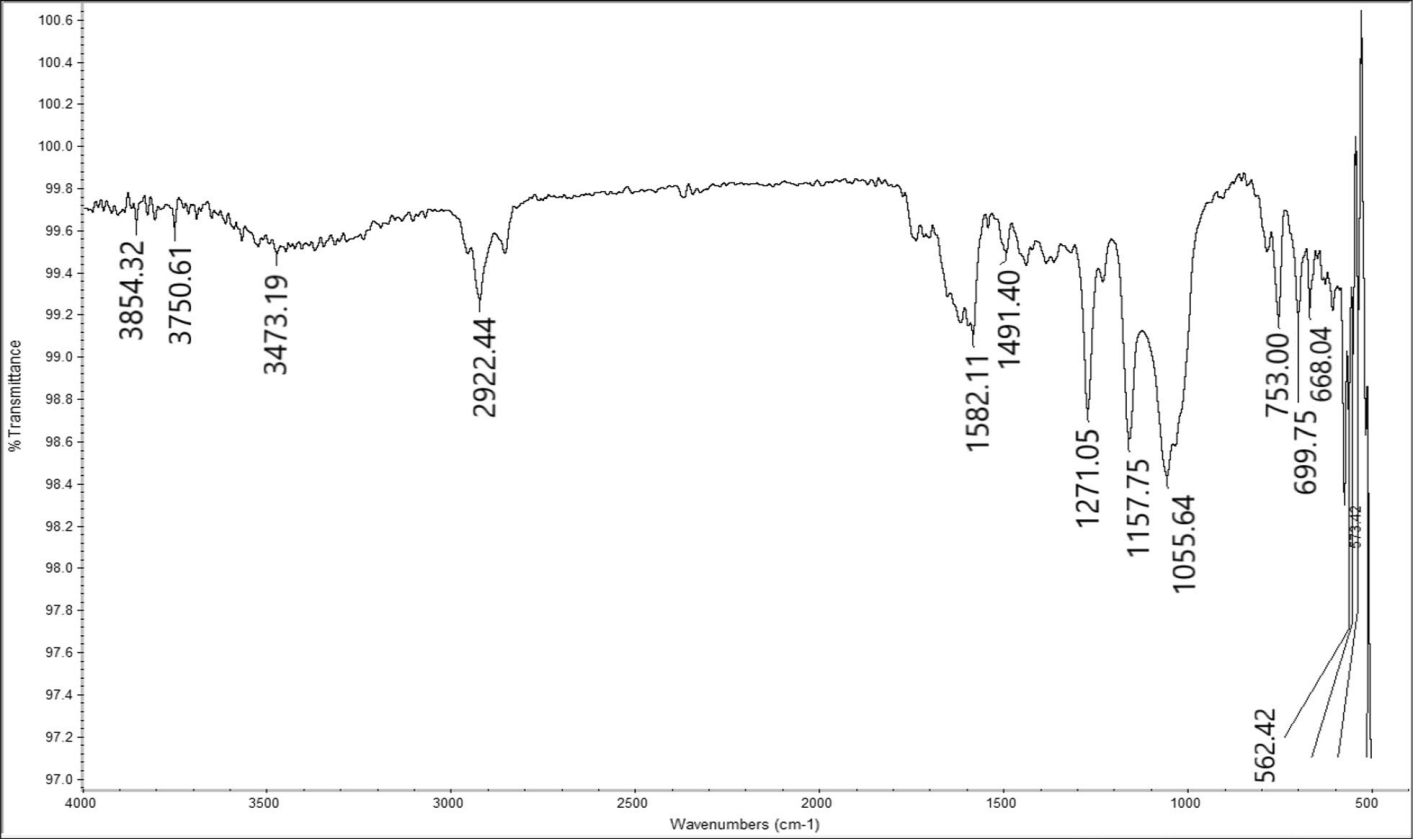
FT-IR spectrum for Cell-β-AN

Cell-AA was reacted with aniline, which undergoes a condensation reaction with the Ketone carbonyl to form Cell-β-AN functionality. The reaction is summarized in Fig 1. The FT-IR spectrum of the Schiff base Cell-β-AN (Fig. 3). The disappearance of the ketone domain at 1709 and the presence of an amine group C-N at about 1271 cm^-1^ is an indication that the amine linkage is formed. The peak at 3473 cm^-1^ is due to N-H vibration of the secondary amine groups. The peak at 1740 cm^-1^ could be attributed to C=O of the ester group. The broad peak at 3430 cm^-1^ could be attributed to the stretching of the hydrogen bonded hydroxyl group (O–H). The amine peak is not sharp, because it overlaps with the OH group peak which has almost the same wavenumber. The carbonyl of the ester group appears at 1740 cm^-1^, IR spectrum shows two bands at about 1157 and 1033 cm^-1^ of C–O–C ring of pyranose ring skeletal and to β-glycosidic linkage vibration, respectively. The adsorption peak at 2922 cm^−1^ is corresponding to symmetric and asymmetric stretching vibration of the C–H bond. The two peaks at about 3010 and 1582 cm^-1^ could be attributed to =C-H and C=C stretching vibrations in an aromatic part of Cell-β-AN as shown in Fig. 3.

**Fig. 4:**
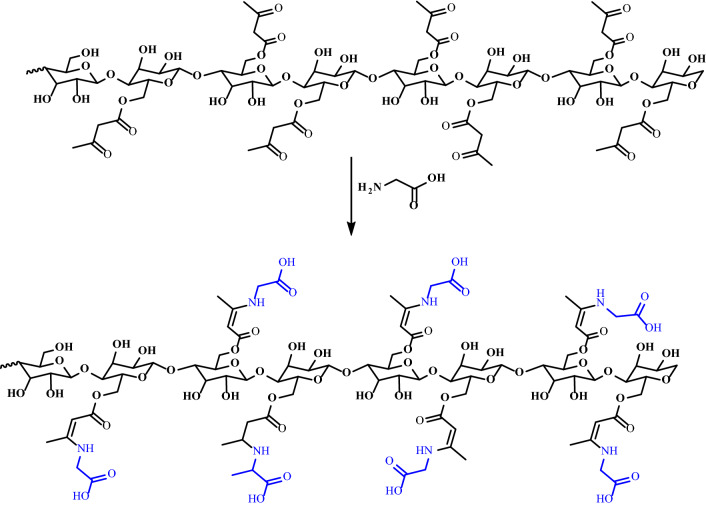
Preparation of cellulose β-aminoacetonate using glycine

**Fig. 5:**
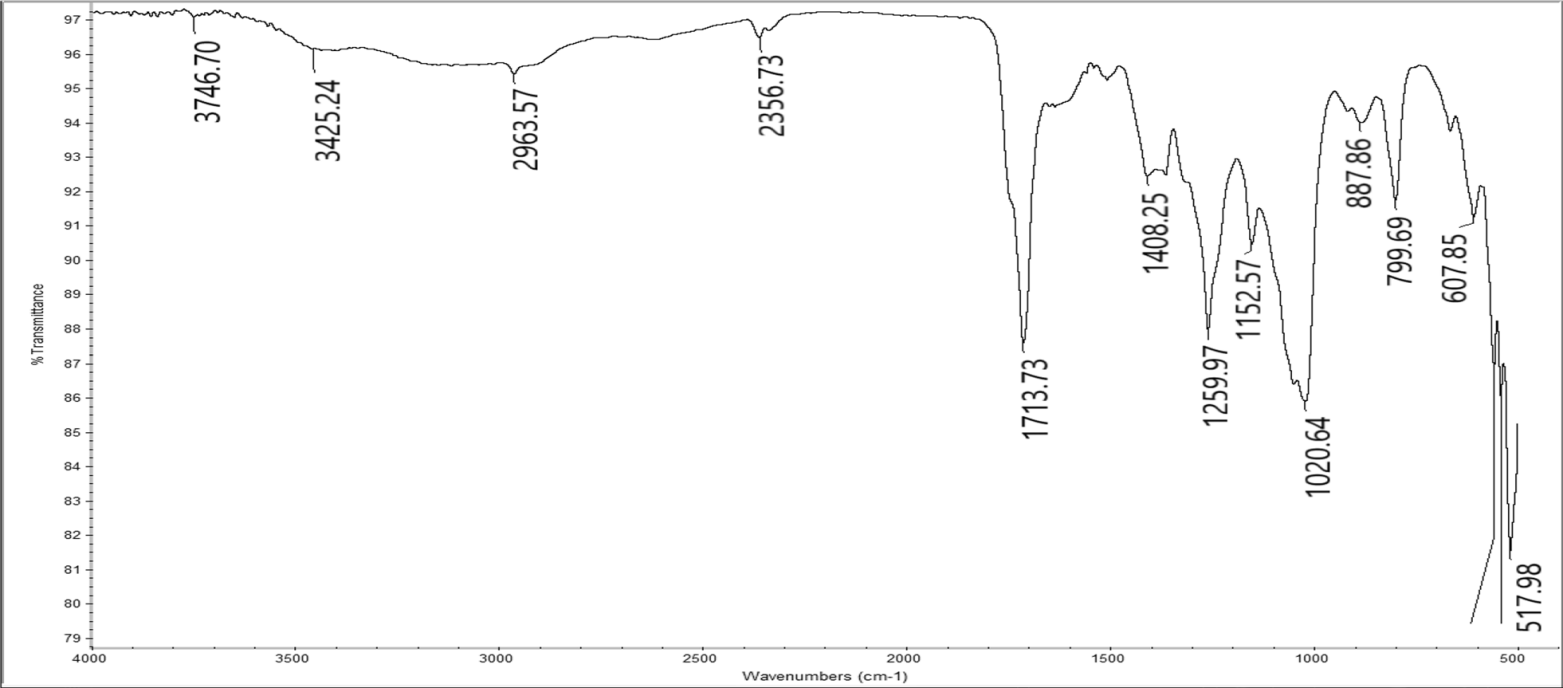
FT-IR spectrum for Cell-β-GL

The cellulose β-glycinocetoester (Cell-β-GL) was produced from reacting cellulose acetoacetate with the amino acid glycine, acetic acid was used as a solvent and a catalyst as proposed in Fig. 4. The FT-IR spectrum of the Cell-β-GL is shown in Fig. 5. The broad strong peak at 1713 cm^-1^ composed of several overlapped peaks that could be attributed to C=O of ester and carboxyl groups. The broad peak at about 3300 cm^-1^ is attributed to the stretching hydrogen bonded hydroxyl group of alcohol and carboxyl.

The IR spectrum also shows three bands at about 1152, 1050 and 1030 cm^-1^ for C-O–C of ester, pyranose ring skeletal and to β-glycosidic linkage vibration respectively. The adsorption peak at 2963 cm^−1^ is corresponding to symmetric and asymmetric stretching vibration of the C-H bond.

**Fig. 6:**
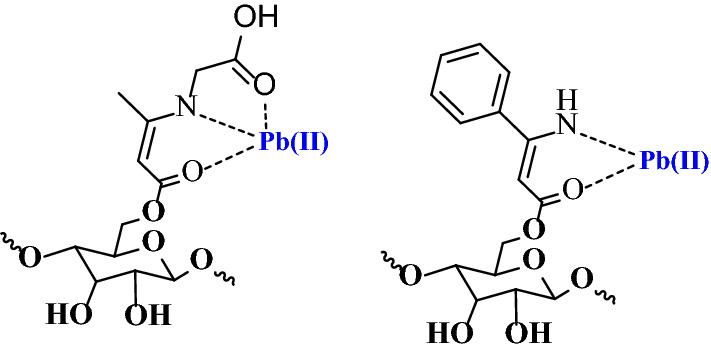
Representative structures show the interaction between Cell-β-GL and Cell-β-AN and the metal ion Pb(II)

The cellulose-based Schiff bases were designed to have a high affinity for various metals. As shown in Fig. 6, the coordination sites bi and tridentate ligand with binding sites contain amines, carbonyl and hydroxyl.

### SEM analysis

**Fig. 7:**
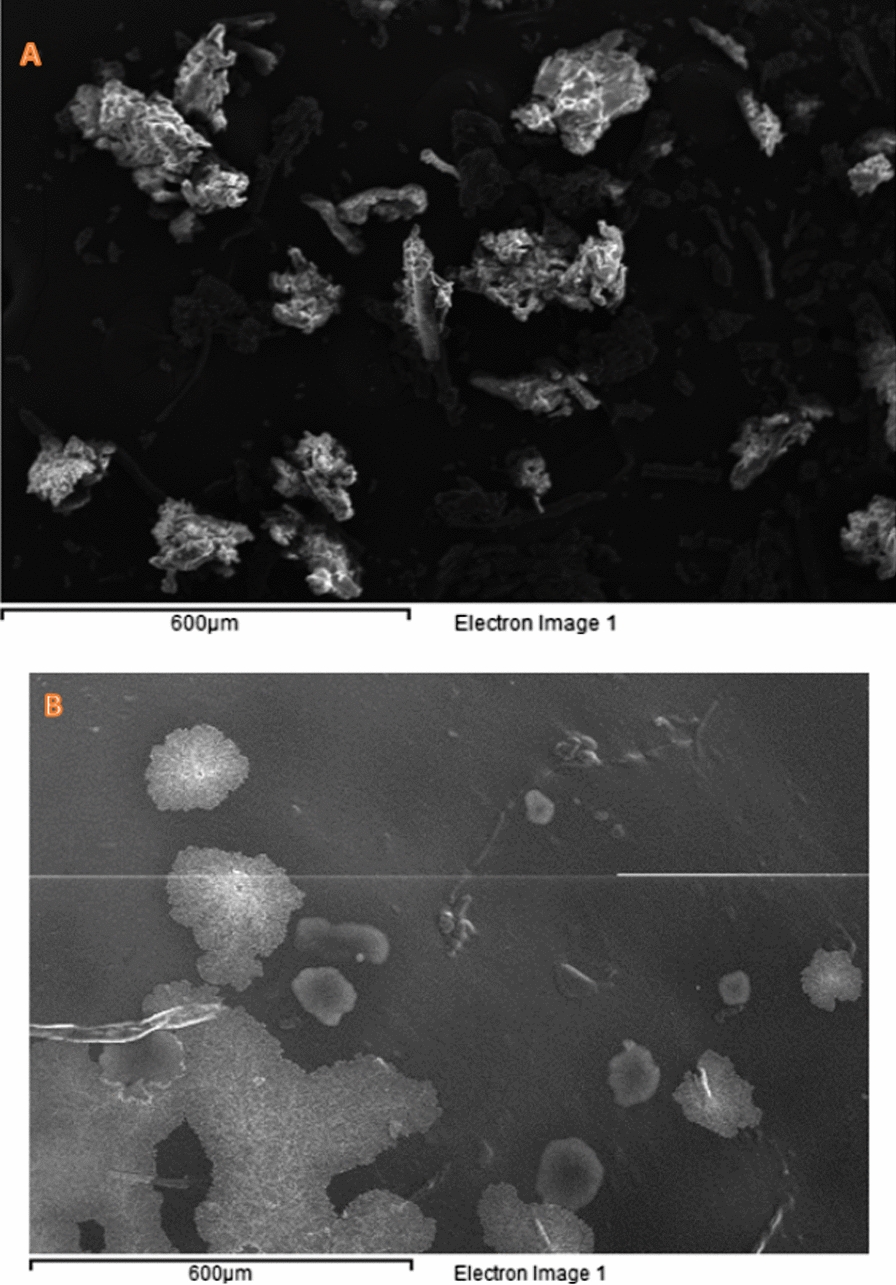
The SEM images of **a** Cell-β-AN and **b** Cell-β-GL at a magnification of 250x 500x, respectively

The SEM images of the two polymers Cell-β-GL and Cell-β-AN are shown in Fig. 7, the images show the surface morphology that appears as a spongy. This explains the high affinity of the polymers for the metal ions.

### Polymer solubility in water

The solubilities of the three polymers in water was determined by suspending 0.5 g of each of the polymers in 50 ml water and stirring for about 6 h. Then collected by suction filtration, dried in an oven at 100 °C, and weighed. Negligible reduction in the weight was noticed.

### TGA analysis and thermal stability

**Fig. 8:**
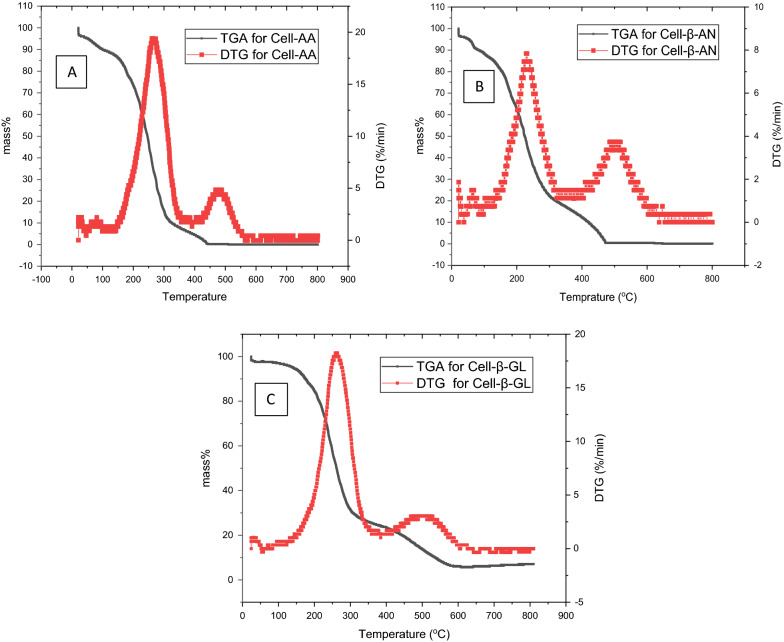
TGA and DTG analysis results of **A** Cell-AA, **B** Cell-β-AN **C** Cell-β-GL polymers

TGA was performed on the three polymers, results are shown in Fig. 8. All polymers show about the same trend, a major drop in the mass appears at 200 °C that could be related to the loss of the pendant group. Complete decomposition started at about 400 °C. The polymers are considered thermally stable since it synthesized mainly for wastewater purification.

### Adsorption of Pb(II)

#### pH effect on adsorption

**Fig. 9:**
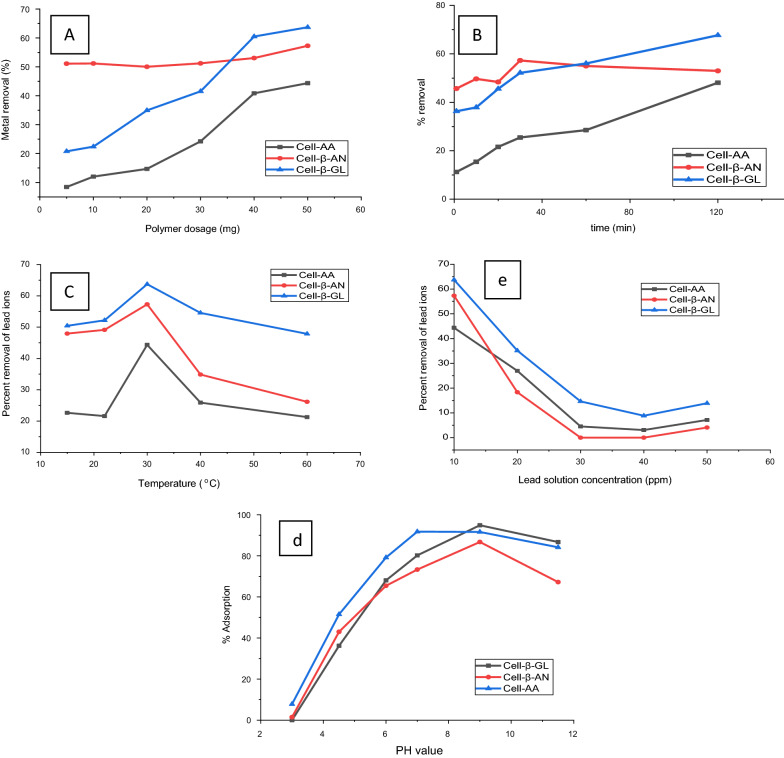
The effect of **a**: adsorbent dose, **b** adsorption time, **c** temperature, **d** pH, and **e** initial ion concentration on the metal removal by the three adsorbents

The effect of the pH value on adsorption efficiency for the three polymers was studied, the other parameters were kept constant (adsorbent dose 40.0 mg, time 30 min, solution volume 10 mL and temperature at 30 °C). The results are shown in Fig 9d. At low pH value (about 3.0) the amine presents in ammonium form (-NR_2_H_2_^+^), also the carboxyl and hydroxyl groups are in protonated form (COOH and OH), so the adsorption efficiency was low. As the pH value increased the amines, carbonyl and hydroxyl groups start to shift to the Lewis base form, causing the hydroxyl, carbonyl and amine to behave as a stronger chelating agent due to the availability of O and N lone pairs of electrons. The highest efficiency was observed at pH 9. At pH value higher than 9, the adsorption efficiency started to decline, this decrease could be related to formation of soluble metal oxide complex which reduced the adsorption efficiency of Pb(II) from the aqueous solution. So, the optimum pH value was selected to be 9.0.

#### Concentration effect on adsorption

The effect of the initial lead ions concentration on adsorption efficiency was also investigated, the other variables being kept constant (pH 4.3, time 30 min, solution volume 10 mL and temperature at 30 °C). The maximum percentage of lead ions removal was about 44.4% by cell-AA, 57.29% by Cell-β-AN and 63.7 % by Cell-β-GL at 10 ppm initial concentration of Pb(II) (Fig. 9e). At concentration higher than 10 ppm the rate of adsorption decreases with increasing the concentration of lead ions. The results show that, at a concentration of 10.0 ppm or lower, there are sufficient binding sites, and the adsorption process is controlled by ion diffusion [60]. As the concentration increases, the availability of the binding sites decreases until the binding site are almost saturated, and the adsorption process is controlled by the adsorbent dosage.

#### Contact time effect on adsorption

The effect of the contact time on %removal was evaluated under conditions of pH 4.3, initial ion concentration 10 ppm, volume of adsorbate 10 ml, adsorption temperature 30 °C and adsorbent dose 50.0 mg. Results are shown in Fig. 9b, the figure shows a sharp increase in the adsorption of Pb(II) after 30 min for all three polymers, which could have related to the availability of plenty of binding sites on the outer surface of the adsorbent. Then a slow increase was observed, the adsorption rate reached equilibrium after about 120 min, so at this period almost all adsorption sites are occupied [60]. A contact time of 30 min was chosen as an equilibrium time for the three polymers.

#### Temperature effect on adsorption

The effect of temperature on the adsorption rate of Pb(II) ions was studied under the conditions shown above at 15, 22, 30, 40 and 60 ^o^C. The highest adsorption rate was found to be at 30 °C. At temperature higher than 30 °C, the percentage of removal tends to decrease as the temperature rises as shown in Fig. 9c. This result is an indication that the adsorption process is spontaneous at low temperature. At high temperature values, over 30 °C, the percentage of metal removal decrease could be related to the kinetic energy of the adsorbed particle on the adsorbent surface increase, which leads to an increase in the possibility of de-complexing from the adsorbent surface.

#### Adsorbent dose effect on adsorption

The effect of adsorbent dosage on %removal is summarized in Fig. 9a. The experiment was performed using various amounts of adsorbents ranging from 5.0 mg to 50.0 mg and 10 ml solutions of Pb(II) with a concentration of 10.0 ppm and a pH value of 4.3. The adsorption time was performed for 30 min at room temperature. The results show that the amount of metal extracted increased by increasing the polymer dosage. The highest removal of about 60.5% was achieved using a 40.0 mg of Cell-β-GL polymers.

#### Desorption studies

**Fig. 10:**
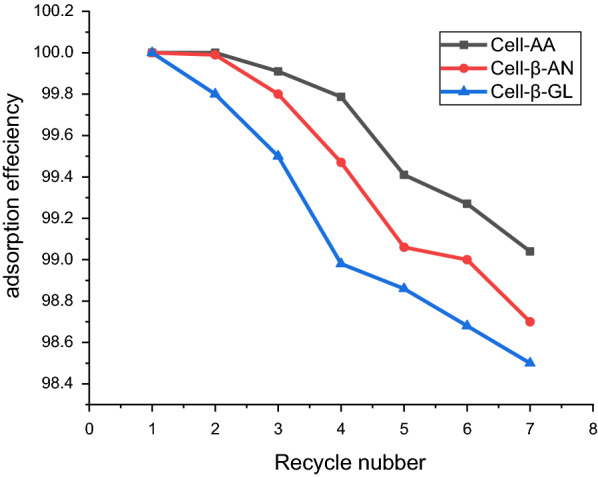
Adsorption efficiency, effect of adsorbent recycling

The regeneration experiment was repeated seven times using the same adsorbent to determine the efficiency of the polymers and the result are shown in Fig. 10. The adsorption efficiency decreases slightly as the number of regeneration cycles increases. In the seventh time, the Cell-AA, Cell-β-GL and Cell-β-AN polymers absorption of lead metals were 99%, 98.5% and 98.7% respectively.

### Wastewater purification from metals

Samples of sewage water were taken from the Beit Dajan wastewater treatment plant in Palestine. Three samples of this water were prepared to be treated with the prepared polymers according to the optimum conditions. The concentrations of the metal ions in each of the sewage samples prior and after using the polymers are summarized in Table 1. Metal ions concentrations were measured using ICP-MS. Excellent efficiency was achieved against some metal ions present in the wastewater samples because polymers contain several coordination sites including hydroxyl, amine, and aromatics groups.

**Table 1:** Percent removal of metal ions present in sewer using the three adsorbents.

Metal Ions	Initial conc. (ppm)	% Removal		
		Cell-AA	Cell-β-AN	Cell-β-GL
Al(III)	12.936	84.539	95.371	82.195
Ba(II)	34.307	94.753	95.737	92.316
B(III)	97.531	98.052	97.575	97.947
Cr(IV)	27.844	89.585	85.270	84.789
Cu(II)	3.773	52.293	90.125	45.593
Fe(III)	205.49	98.686	99.745	98.201
Pb(II)	7.473	87.957	61.165	89.476
Mn(II)	19.822	87.388	90.582	84.893
Ni(II)	4.139	85.504	59.839	72.363
V(III)	1.973	8.768	59.253	64.587
Zn(II)	13.07	68.631	95.371	82.195

#### .

### Adsorption analysis

#### Isotherm

Langmuir (Eq. 3) and Freundlich isotherm (Eq. 5) models were applied to investigate the adsorption equilibrium between Pb(II) ion solution and the three adsorbents [20]. Both models were used to assess the metal ion dispersion on the adsorbent surface at the equilibrium stage. The value of the correlation coefficients, R^2^ (Eq. 4) can lead to the type of isotherm model of the adsorption process. The *R*_*L*_ ratio was defined as a dimensionless quantity indicating that sorption is favorable or not, since if the value of R_L_ is higher than 1, this indicates that the adsorption is unfavorable. However, when the R_L_ value is between 1 and 0, this indicates favorable adsorption, whereas when R_L_ = 1 indicates the presence of linear adsorption [20].

3 $$ \frac{{C_{e} }}{{Q_{e} }} = \frac{1}{{q_{\max } }}\,C_{e} + \frac{1}{{q_{\max } K_{L} }} $$

Where C_e_ represents the equilibrium concentration of the adsorbate (mg/L), Qe is the amount of the adsorbate adsorbed per unit mass of cellulose-based polymers at equilibrium (mg/g), q_max_ is the adsorption capacity equilibrium (mg/g), and K_L_ is usually, the Langmuir affinity constant (L/mg).

4 $$ R_{L} = \frac{1}{{1 + K_{{L C_{0} }} }} $$

C_o_ is the initial adsorbate concentration.

5 $$ {\text{In}}\,\left( {q_{e} } \right) = {\text{In}}\,k_{F} + \frac{1}{n}{\text{In }}C_{e} $$

K_F_ is the Freundlich constant that deals with adsorption capacity (mg/g) and n is the heterogeneity coefficient which leads to how favorable the adsorption process (g/L).

**Fig. 11:**
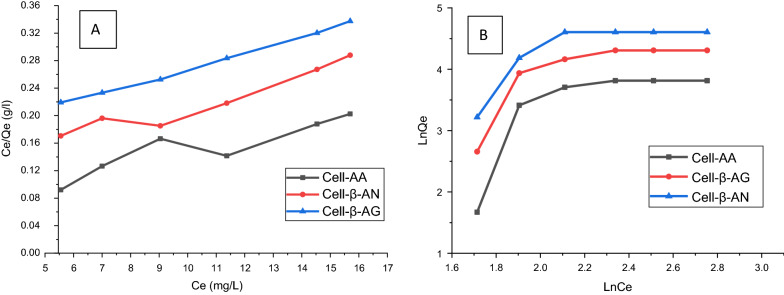
**A** Langmuir adsorption model and **B** Freundlich adsorption model of Pb(II) ions on three adsorbents

Figure 11 summarizes all adjustment parameters. The correlation coefficients of the Freundlich isotherm model is lower for Cell-AA while it is higher for Cell-β-AG and Cell-β-AN than those of the Langmuir isotherm model (Table 2), reflecting that the adsorption of Pb(II) ions obey the Freundlich isotherm model for Cell-β-AG and Cell-β-AN and Langmuir isothermal model for Cell-AA. The results indicate a single-layer adsorption behavior with a heterogeneous energy distribution of the active sites along with the interactions between adsorbent and adsorbate. However, in the case of Cell-AA polymer the Pb(II) cation are distributed equally and homogeneously across the porous surfaces of the cellulose based polymers [61].

**Table 2:** Langmuir and Freundlich parameters for the adsorption of Pb(II) ions by cellulose-based polymers

	Pb(II)			
	Cell-AA	Cell-β-AG	Cell-β-AN	
Langmuir isotherm				
Q^0^ (mg/g)	2.4587	2.1256	2.1254	
K_L _(L/mg)	0.1524	0.1202	0.1965	
R^2^	0.9625	0.8958	0.8548	
Freundlich isotherm				
1/n	1.2154	0.9587	1.2548	
K_F _(L/mg)	16.325	23.3254	17.325	
R^2^	0.8536	0.9621	0.93254	

The separation factor R_L_, which has been calculated for different quantities of adsorbent, ranges from 0< R_L_<1 (Table 2). This reflects the high degree of affinity of the three adsorbents for the studied metal ions.

#### Adsorption kinetics

The kinetic of the adsorption of metal ion Pb(II) by the three adsorbents was evaluated using the kinetic models: pseudo-first order (Eq. 6) and pseudo-second order models (Eq. 7) [62]. Weber and Morris developed Eq. 8 describing the intraparticle diffusion [62].

6 $$ {\text{ ln}}(q_{e} - q_{t} ) = \ln q_{e} - {\text{K}}_{{1}} {\text{t}} $$

7 $$ \frac{1}{{{\text{q}}_{{\text{t}}} }} = \frac{1}{{{\text{K}}_{{2{\text{q}}_{e}^{2} }} }} + \frac{{\text{t}}}{{{\text{q}}_{e} }} $$

8 $$ {\text{Q}}_{{\text{t}}} = {\text{ K}}_{{{\text{id}}}} {\text{t}}^{{{1}/{2}}} + {\text{ Z}} $$

where Q_t_ (mg g^-1^) is adsorption capacity at any time t, k_id_ (mg/g min^1/2^) is the intraparticle diffusion rate constant, and Z (mg/g) is a constant proportional to the thickness of the boundary layer.

**Fig. 12:**
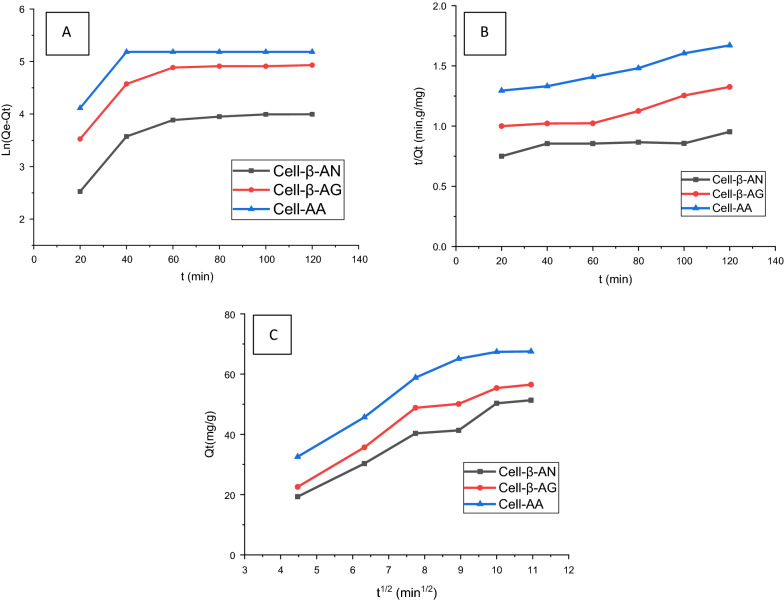
**A** Pseudo first-order model **B** Pseudo-second order model and **C** Intra-particle diffusion model for the adsorption of Pb(II) ions onto cell-AA, cell-β-AN, and cell-β-AG at various concentrations

Table 3 and Fig. 12 summarize the values of all parameters obtained using the above equations. The plots of Ln (q_e_-q_t_) versus t (Fig. 12A) provide the value of K_1_, whereas the values of K_2_ and the adsorption capacity q_e_ were derived from the slope and intercept of the plot of t/Qt versus t (Fig. 12B), while K_id_ and Z were deduced by tracing Qt vs t^1/2^ (Fig. 12C).

**Table 3:** The pseudo-second-order model for adsorption of Pb(II) ions onto cell-AA, cell-β-AN, and cell-β-AG

	Cell-AA				Cell-β-AN			Cell-β-AG				
	K_2_ (g/mg.min)	Q_cal_ (mg/g)	R^2^	K_2_ (g/mg.min)		Q_cal_ (mg/g)	R^2^		K_2_ (g/mg.min)	Q_cal_ (mg/g)	R^2^	
Pb(II)	0.3356	427.3254	0.9885	0.4325		548.3224	0.9750		0.465	632.2134	0.9887	

	Cell-AA			Cell-β-AN			Cell-β-AG		
	K_id_	Z	R^2^	K_id_	Z	R^2^	K_id_	Z	R^2^
Pb(II)	0.1625	5.9021	0.9402	0.1503	5.8507	0.9514	0.1844	5.6977	0.9465

Parameters explain the intra-particle diffusion of Pb(II) ions onto cell-AA, cell-β-AN, and cell-β-AG.

The experimental results show that the correlation coefficient (R^2^) for the pseudo-second order kinetics model (0.91 to 0.973) was greater than the value obtained by pseudo-first order (0.891). Also, the qe values (2.675, 15.252, 20.856 mg/g) which are close to the experimental qe values (2.133, 13.91, 18.786 mg/g) for the three polymers Cell-AA, Cell-β-AG and Cell-β-AN, respectively, indicating that the adsorption process follows the pseudo-second order model for adsorption of Pb on the surfaces of the three polymers obey the pseudo-second order (Table 3 and Fig. 12B).

From (Fig. 12C) (Qt *vs.* t^1/2^) K_id_ and Z were calculated and reported in Table 3. All graphs plotted in Fig. 16 didn’t cross the origin, indicating the occurrence of more than one rate-limiting process.

Based on initial graphs linearity presented in Fig. 12B it can be conclude that, at the outset of the adsorption process, the adsorption of Pb(II) on the three polymers takes place initially by an instantaneous adsorption step (on the external surface), which caused a chemical complexation between the metal ions and functional groups, COOH, NR_2_ and the OH [21, 24, 63–70]. The other steps were also linear, showing a progressive adsorption of Pb(II) ions and the step of limiting intraparticle diffusion rate.

The results presented in Table 3 reveal that the Z values reflect an expansion in the upper layer of the adsorbent and a decrease in the outer mass transfer although the inner mass transfer potential was increasing. The energy of activation of the adsorption process was computed at 298 and 323 K according to Eq. 8.

These findings are important for understanding how temperature influences adsorption performance of three polymers. The activation energy computed was nearly zero, suggesting a spontaneous adsorption process.

#### Thermodynamics study

**Fig. 13:**
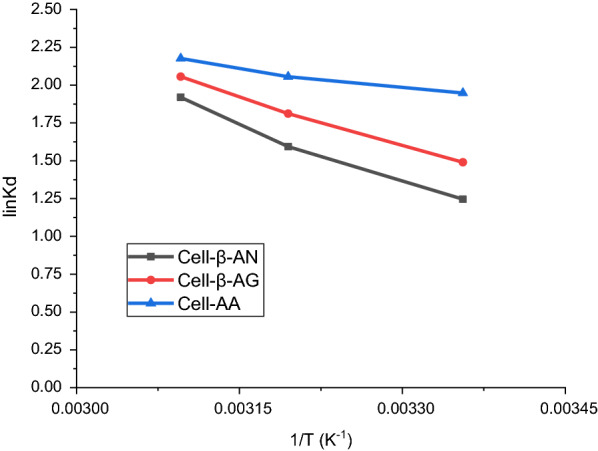
Adsorption thermodynamics of Pb(II) ions onto cell-AA, cell-β-AN, and cell-β-AG

The thermodynamic parameters free energy, standard enthalpy, and standard entropy for adsorption of Pb(II) by the three polymers were calculated using the following equations [52]. The aim of this study is to understand the spontaneity and the nature of adsorption.

9 $$ {\text{K}}_{{\text{c}}} = {\text{ C}}_{{{\text{ads}}}} /{\text{C}}_{{\text{e}}} $$

10 $$ \Delta {\text{G}}^{0} = \, - {\text{RTlnK}}_{{\text{c}}} $$

11 $$ {\text{In}}\,K_{S} \frac{\Delta S}{R} - \frac{\Delta H}{{RT}} $$

where K_c_ is an apparent constant of the thermodynamics; and C_ads_ and C_e_ are respectively the amount adsorbed at equilibrium (mg/L) and concentration of metal ion in the solution (mg/L), R is the universal gas constant (8.314 J/mol K); T is the solution temperature [54]. The (ΔG^0^) (J mol^-1^) value was determined according to Eq. 10. The ln K_s_ *vs.* 1/T was mapped as illustrated in Fig. 13, the slopes and crossings were utilized to determine various thermodynamics parameters as shown in Table 4.

**Table 4:** Thermodynamic parameters for the adsorption of Pb(II) ions onto cell-AA, cell-β-AN, and cell-β-AG

Pb(II)			
	∆G° (KJ/mol)	∆H° (KJ/mol)	∆S° (J/K.mol)
Cell-AG	− 17.2525	13.20211	74.92155
Cell-β-AG	− 18.2314		
Cell-β-AG	− 18.8021		

The value obtained for ΔS^0^ and ΔH^0^ are positive, whereas the entropy raised at the solid/solution interface induced as a result of the adsorption process. The findings further indicate that, the free energies for the three polymers were negative reflecting a spontaneous process of adsorption at various temperatures.

The results indicate that the metal removal occurs at various stages. In the first stage, metal ions migrate from the solution to the outer surface of the adsorbent, then diffuse across the boundary-layer to the outer surface of the adsorbents, followed by coordination of metals ions at the binding sites on the adsorbent surface, and lastly, intra-particle diffusion and adsorption of ions across the adsorbent particles.

### Monte Carlo and molecular dynamic simulations

Recognizing the adsorbate molecules' preferred adsorption arrangement on the Cell-β-AN or GL surface is crucial for determining the various energy outputs.

The interaction of the adsorbate ions Pb^2+^with the modified cellulose surface enables the calculation of this method's adsorption energetics. This is performed quantitatively by use the equation below to determine the adsorption energy (Eads) [71–77] :

12 $$  E_{{adsorption}}  = \,E_{{Pb\left( {II} \right)/Cell - \beta  - GLorAN}}  - \left( {Cell - \beta  - GLor{\mkern 1mu} AL + Pb\left( {II} \right)} \right) $$

where ${E}_{Pb(II)/Cell-\beta -GL orAL}$ is the total energy of the simulated adsorption system, $Cell-\beta -GL orAN$ and $Pb(II)$ is the total energy of the adsorbate ions.

**Fig. 14:**
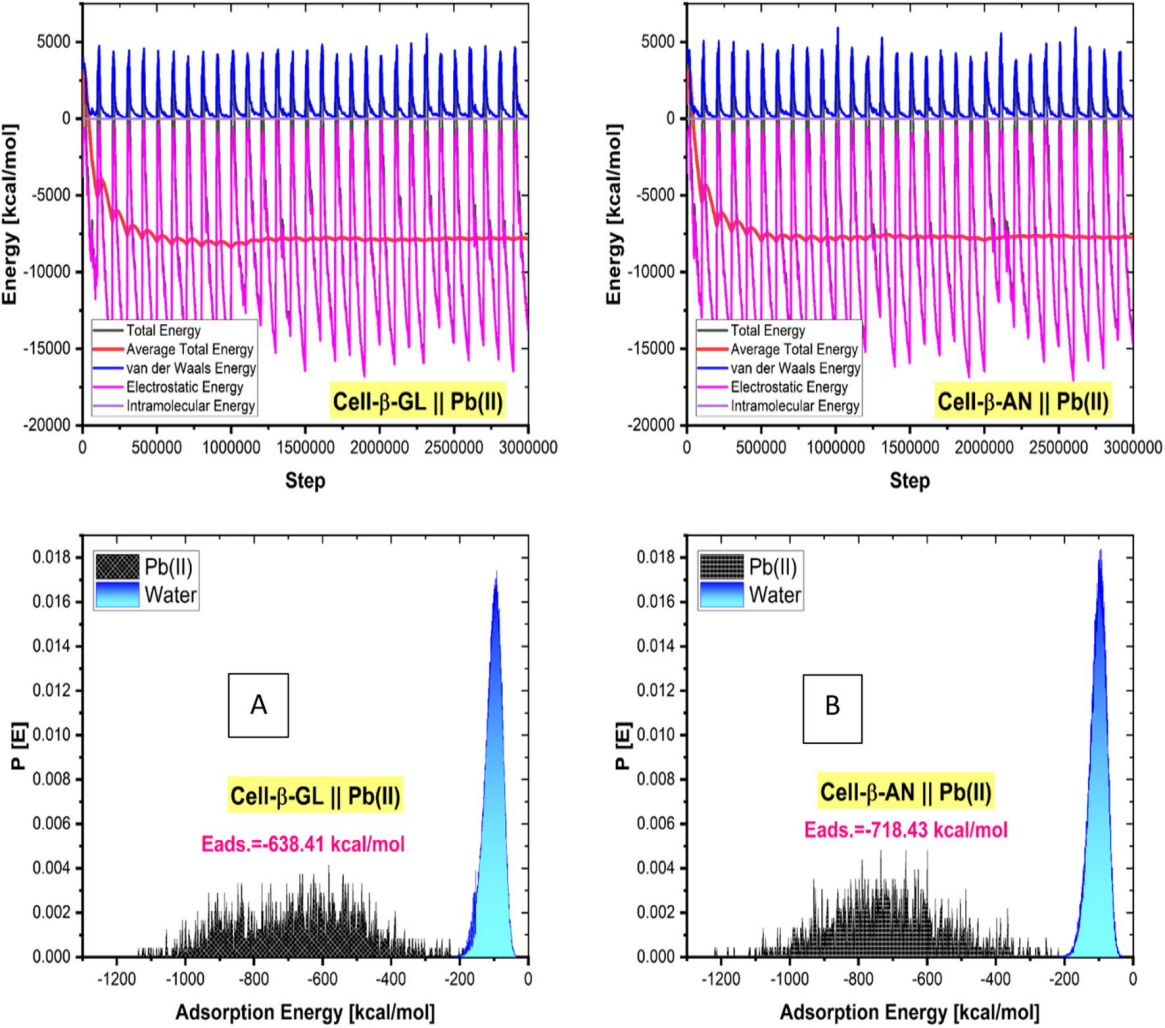
**A** Different energy terms during the exploration of random MC configurations (3 000 000) and **B** Probability of the adsorption energy distributions during MC for the adsorbate ions onto modified cellulose surface

Figure 14 shows the energy terms and the energy evolution during MC for the most sable or low energy adsorption sites of adsorbates in the vicinity of the modified cellulose surface obtained through an excessive number of randomly generated Monte Carlo calculations.

The experimental findings are supported by a strikingly superior negative value of Eads of the adsorbate ions onto the both modified cellulose surface.

**Fig. 15:**
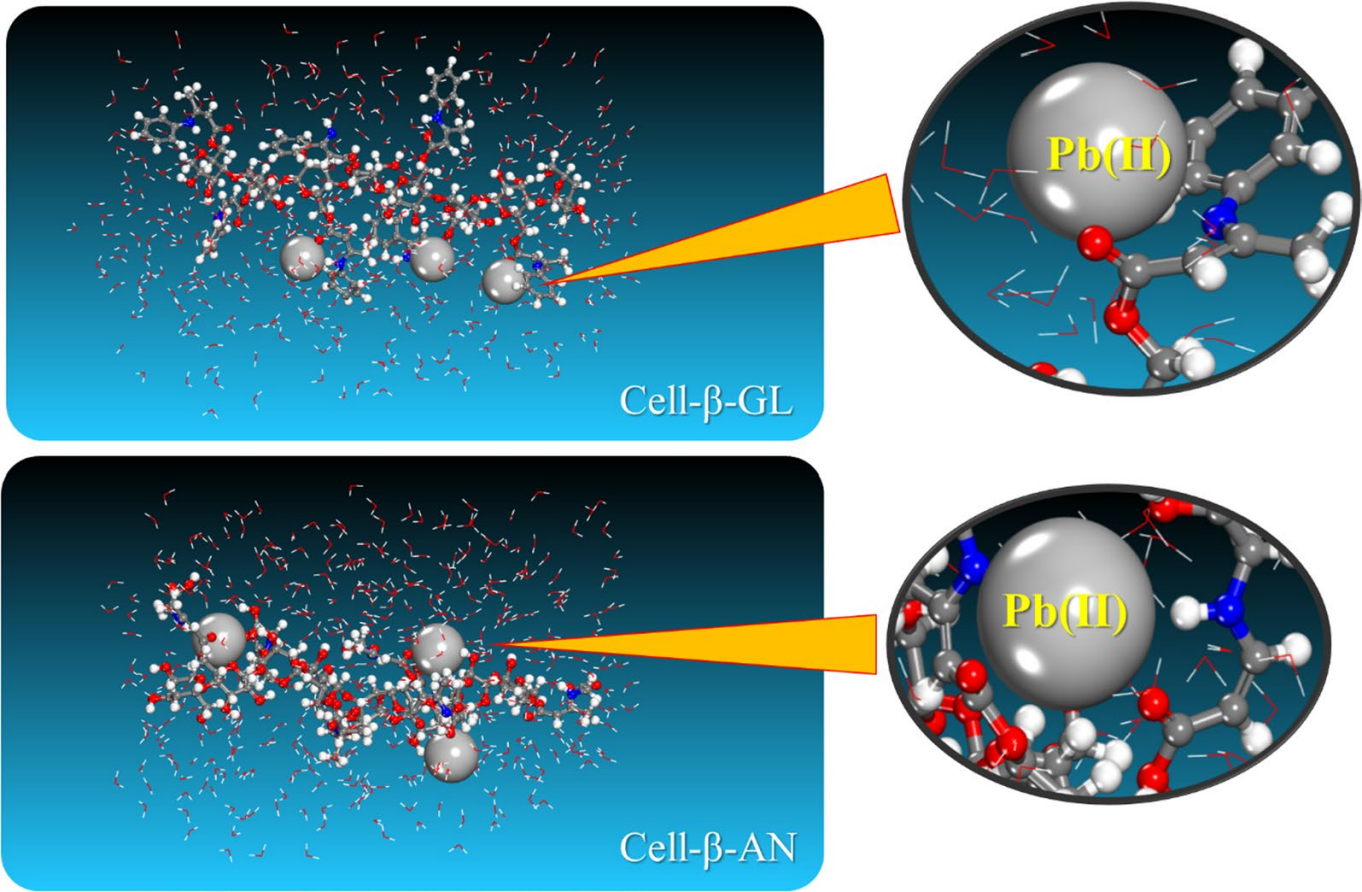
Lowest energy configurations of Pb Pb^2+^ ions onto the corresponding modified cellulose surfaces as obtained from MD

The method for measuring and imaging the dynamics of inhibitor adsorption on the materials surface is used in MD simulation. Fig. 15 shows the adsorbate ions final structure on the modified cellulose surfaces.

**Fig. 16:**
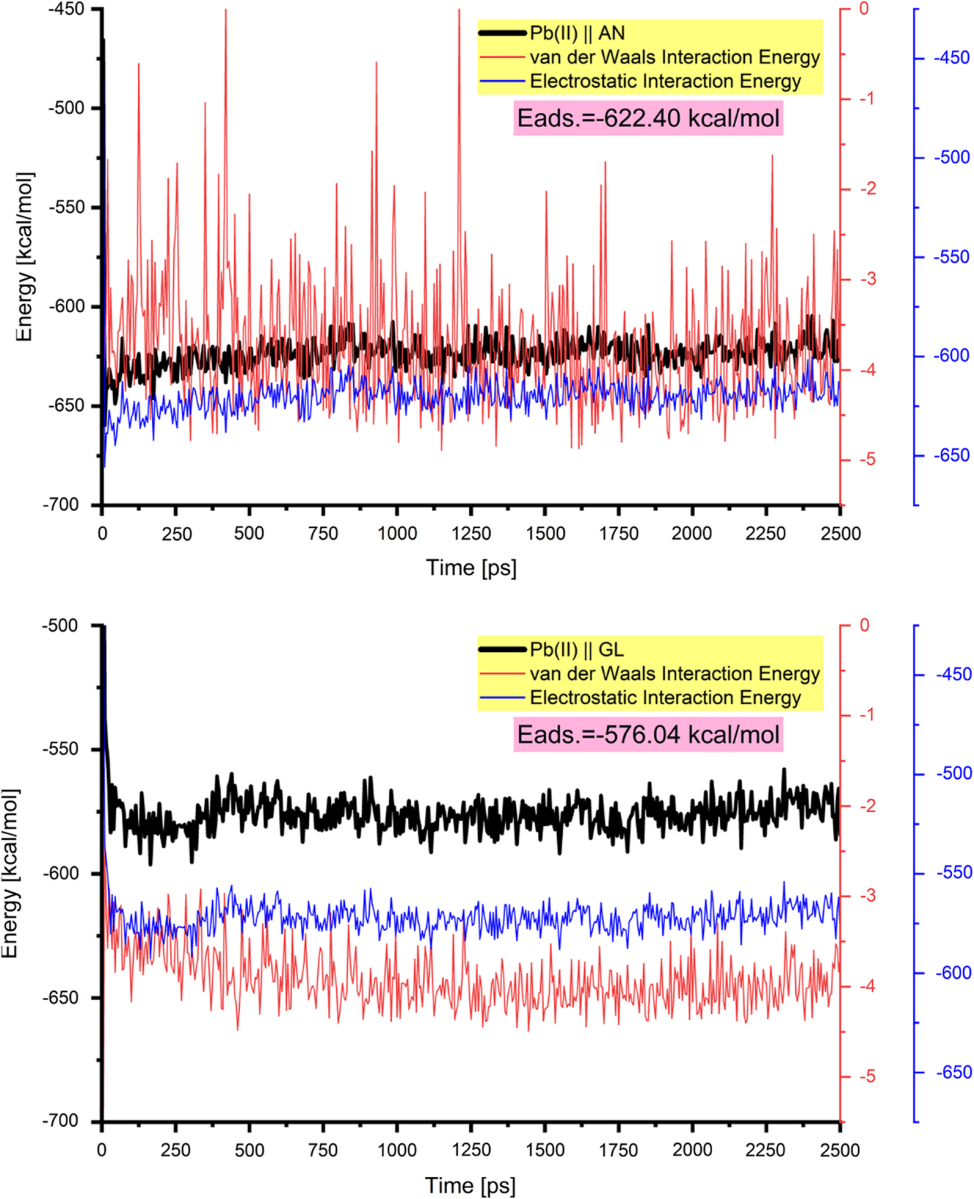
The change of the adsorption energy (and corresponding energy terms) for the Pb^2+^ions onto the modified cellulose surfaces obtained during the MD

As can be seen in Fig. 16, where the adsorption energy of the lead ions is calculated over the course of the entire trajectory, the adsorption of the lead ions occurs spontaneously (as indicated by the relatively high adsorption energy values), and the results are consistent with those obtained experimentally [77, 78]. The mean of the adsorption energy is calculated after the system equilibration (last 2000 ps of the MD trajectory). The interaction is based mostly on the electrostatic one with a contribution through van der Waals forces.

The relatively small changes of the adsorption energy (black line) energy as observed in Fig. 16, indicate that the system has reached the lowest energy.

To better understand this interaction DFT calculations involving a monomeric system of modified cellulose were performed.

**Fig. 17:**
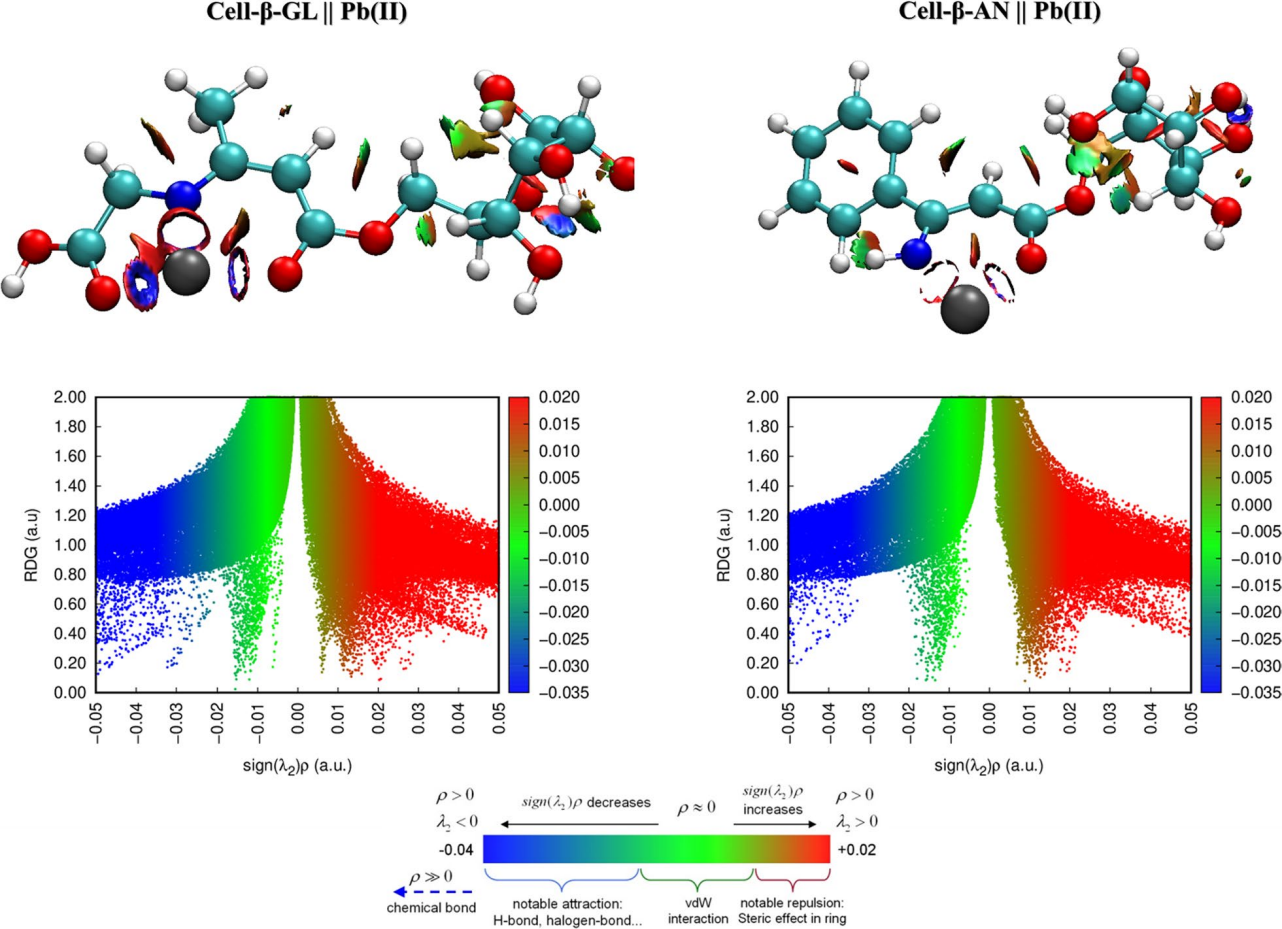
Noncovalent interaction surfaces and the plot of RDG vs sign(λ)ρ for the van der Waals interactions among the Pb^2+^ions and the modified cellulose moieties

The assessment of the interaction nature amid the Pb^2+^ions and the modified cellulose structures is performed via the NCI surface plot and the reduced density gradient (RDG) vs. sign (λ) (Fig. 17) [79, 80]. The greenish-blueish colored surface and the spikes with negative sign (λ) values in the 2D NCI plot support that the van der Waals interactions are presented in the formed structures.

**Fig. 18:**
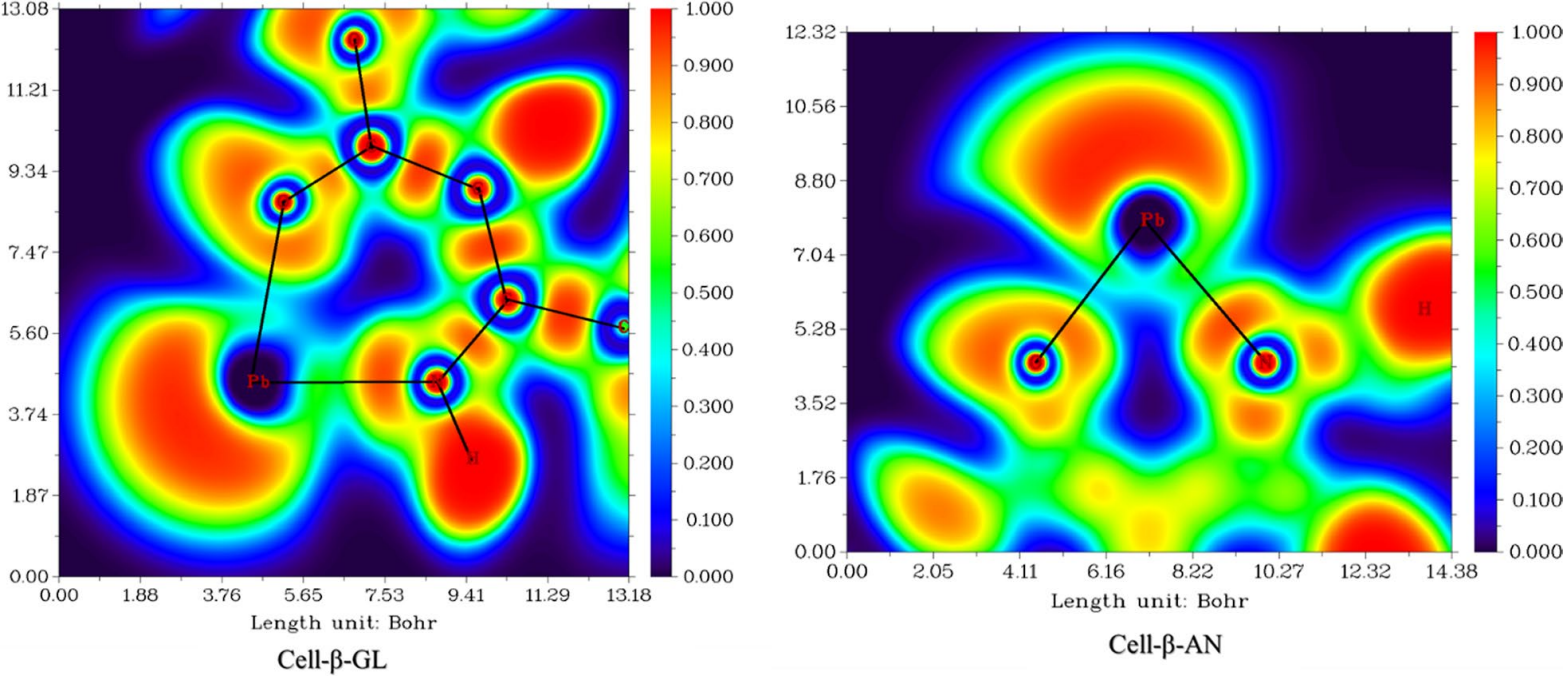
Electron localization function (ELF) analysis of the “bonding” between Pb^2+^ions and the side groups of the modified cellulose

The ‘bonding’ interaction among the Pb(II) ions and the side groups of the modified cellulose is discernible via the ELF analysis, where the low values of ELF indicate the low degree of covalence of these formed bonds [79]. This "binding" is also evident when Mayer's binding order analysis is applied as shown in Fig. 18 and Table 5.

**Table 5:** Mayer bond order for selected bonding atoms in the Pb^2+^/ modified cellulose structures.

System	Bonding atoms	Mayer bond order
Pb(II)|| GL	Pb-O	0.313
	Pb-O	0.412
	Pb-N	0.197
Pb(II)|| AN	Pb-O	0.339
	Pb-N	0.507

The Mayer bond order ruptures the electron density in such a mode that the degree of bonding is calculated in a modest way, where a perfectly fulfilled double bond has a value of 2, a triple bond has a value of 3, and so on as shown in Table 5 [80]. The bond order values point to that the interaction of the Pb^2+^ions is moderately strong paralleled to other types of coordinative binding [81].

## Conclusion

Cellulose used in this study was extracted from olive industry solid waste, it was successfully functionalized with the pendant group β-amino ester by first introducing 1,3-dicarbonyl to the cellulose repeat unit then reacting it with aniline and the amino acid glycine. The structures of the target polymers were identified by FT-IR spectroscopy and other techniques. The prepared three polymers showed excellent efficiency toward removal of toxic metal ions from wastewater. The optimum value of various parameters (contact time, pH value, adsorbent dose, temperature, and initial concentration of lead ion) that lead to highest adsorption efficiency were determined. The adsorption mechanism follows the Langmuir isotherm model. Kinetic data revealed that the adsorption of Pb(II) obeys the pseudo second order. Thermodynamic study showed negative Gibbs free energies, indicating a spontaneous adsorption process of Pb(II) by the three polymers. Theoretical calculation using Monte Carlo (MC) and Molecular Dynamic (MD) simulation models were conducted to confirm the experimental results of strong interaction and spontaneous adsorption between Pb(II) and the functional groups on the cellulose polymers. The polymers quantitatively removed metal ions present in a real sample of sewage and showed good desorption properties.

## Data Availability

Adequate and clear descriptions of the applied materials and tools are provided in the materials and method section of manuscript. In addition, the obtained data is clearly justified by mentioning the figures and tables in the manuscript.

## References

[1] Goel PK (2006). Water pollution: causes, effects and control.

[2] Shawai SAA, Muktar HI, Bataiya AG, Abdullahi II, Shamsuddin IM, Yahaya AS, Suleiman M (2017). A review on heavy metals contamination in water and soil: effects, sources and phytoremediation techniques. Int J Miner Process Extr Metall.

[3] Bharathy N (2018). Water pollution and water quality standards for livestock. Int J Sci Environ Technol.

[4] Junior ACG, Schwantes D, Junior EC, Zimmermann J, Coelho GF (2021). Adsorption of Cd (II), Pb (II) and Cr (III) on chemically modified *Euterpe oleracea* biomass for the remediation of water pollution. Acta Sci Technol.

[5] da Paz Schiller A, Ferronato MC, Schwantes D, Gonçalves AC, Barilli DJ, Manfrin J (2019). Influence of hydrological flows from tropical watersheds on the dynamics of Cu and Zn in sediments. Environ Monit Assess.

[6] Masindi V, Muedi KL, Saleh HE-DM, Aglan RF (2018). Environmental contamination by heavy metals. Heavy metals.

[7] Sud D, Mahajan G, Kaur MP (2008). Agricultural waste material as potential adsorbent for sequestering heavy metal ions from aqueous solutions–A review. Bioresour Techno.

[8] Abdel-Ghani NT, Hefny M, El-Chaghaby GA (2007). Removal of lead from aqueous solution using low cost abundantly available adsorbent. Int J Environ Sci Technol.

[9] Peng H, Guo J (2020). Removal of chromium from wastewater by membrane filtration, chemical precipitation, ion exchange, adsorption electrocoagulation, electrochemical reduction, electrodialysis, electrodeionization, photocatalysis and nanotechnology: a review. Environ Chemis Lett.

[10] Taseidifar M, Makavipour F, Pashley RM, Rahman AM (2017). Removal of heavy metal ions from water using ion flotation. Environ Technol Innov.

[11] Silwamba M, Ito M, Hiroyoshi N, Tabelin CB, Hashizume R, Fukushima T, Ishizuka M (2020). Recovery of lead and zinc from zinc plant leach residues by concurrent dissolution-cementation using zero-valent aluminum in chloride medium. Metals.

[12] Mcdonald CW, Bajwa RS (1977). Removal of toxic metal ions from metal-finishing wastewater by solvent extraction. Sep Sci.

[13] Bashir A, Malik LA, Ahad S, Manzoor T, Bhat MA, Dar GN, Pandith AH (2019). Removal of heavy metal ions from aqueous system by ion-exchange and biosorption methods. Environ Chemis Lett.

[14] Tsitonaki A, Petri B, Crimi M, MosbÆK H, Siegrist RL, Bjerg PL (2010). In situ chemical oxidation of contaminated soil and groundwater using persulfate: a review. Crit Rev Environ Sci Technol.

[15] Thaçi BS, Gashi ST (2019). Reverse osmosis removal of heavy metals from wastewater effluents using biowaste materials pretreatment. Pol J Environ Stud.

[16] Alqadami AA, Naushad M, ALOthman, Z. A., Alsuhybani, M., & Algamdi, M.  (2020). Excellent adsorptive performance of a new nanocomposite for removal of toxic Pb (II) from aqueous environment: adsorption mechanism and modeling analysis. J Hazard Mater.

[17] Sen TK (2017). Air, gas, and water pollution control using industrial and agricultural solid wastes adsorbents.

[18] Afroze S, Sen TK (2018). A review on heavy metal ions and dye adsorption from water by agricultural solid waste adsorbents. Wat Air Soil Poll.

[19] Demirbas A (2008). Heavy metal adsorption onto agro-based waste materials: a review. J Hazard Mater.

[20] Anirudhan TS, Sreekumari SS (2011). Adsorptive removal of heavy metal ions from industrial effluents using activated carbon derived from waste coconut buttons. J Environ Sci.

[21] Moa J, Yanga Q, Zhang N, Zhang W, Zheng Y, Zhang Z (2018). Review on agro-industrial waste (AIW) derived adsorbents for water and wastewater treatment. J Environ Manag.

[22] Şenol ZM, Gül ÜD, Gurbanov R, Şimşek S (2021). Optimization the removal of lead ions by fungi: explanation of the mycosorption mechanism. J Environ Chem Eng.

[23] Şenol ZM, Gül ÜD, Şimşek S (2019). Assessment of Pb2+ removal capacity of lichen (Evernia prunastri): application of adsorption kinetic, isotherm models, and thermodynamics. Environ Sci Poll Res.

[24] Jodeh S, Hamed O, Melhem A, Salghi R, Jodeh D, Azzaoui K, Benmassaoud Y, Murtada K (2018). Magnetic nanocellulose from olive industry solid waste for the effective removal of methylene blue from wastewater. Environ Sci Poll Res.

[25] Chakraborty R, Asthana A, Singh AK, Jain B, Susan ABH (2020). Adsorption of heavy metal ions by various low-cost adsorbents: a review. Int J Environ Anal Chemis.

[26] Gupta S, Babu BV (2009). Utilization of waste product (tamarind seeds) for the removal of Cr(VI) from aqueous solutions: equilibrium, kinetics, and regeneration studies. J Environ Manage.

[27] Muhammad RR, Nor AY, Mohammad JH, Norazowa I, Faruq M, Sazlinda K, Hamad A (2018). A iminodiacetic acid modified kenaf fiber for waste water treatment. Int J Biol Macromol.

[28] Karnitz O, Gurgel LVA, de Melo JCP (2007). Adsorption of heavy metal ion from aqueous single metal solution by chemically modified sugarcane bagasse. Bioresour Technol.

[29] Doan HD, Lohi A, Dang VBH, Dang-Vu T (2008). Removal of Zn^+2^ and Ni^+2^ by adsorption in a fixed bed of wheat straw. Process Safe Environ Prot.

[30] Acar FN, Eren Z (2006). Removal of Cu(II) ions by activated poplar sawdust (Samsun clone) from aqueous solutions. J Hazard Mater.

[31] Vieira MGA, de Almeida Neto AF, Da Silva MGC, Carneiro CN, Melo Filho AA (2014). Adsorption of lead and copper ions from aqueous effluents on rice husk ash in a dynamic system. Braz J Chem Eng.

[32] Hamed O, Fouad Y, Hamed EM, Al-Hajj N (2012). Cellulose powder from olive industry solid waste. BioResources.

[33] Hamed OA, Jodeh S, Al-Hajj N, Hamed EM, Abo-Obeid A, Fouad Y (2015). Cellulose acetate from biomass waste of olive industry. J Wood Sci.

[34] Klamt A (2005). COSMO-RS: from quantum chemistry to fluid phase thermodynamics and drug design.

[35] Klamt A (2018). The COSMO and COSMO-RS solvation models. Wiley Interdiscip Rev Comput Mol Sci.

[36] Berisha A (2019). Interactions between the aryldiazonium cations and graphene oxide: a DFT study. J Chemis.

[37] Molhi A, Hsissou R, Damej M, Berisha A, Bamaarouf M (2021). Performance of two epoxy resins against corrosion of C38 steel in 1M HCl: electrochemical, thermodynamic and theoretical assessment. Int J Corros Scale Inhib.

[38] Neese F (2018). Software update: the ORCA program system, version 40. Wiley Interdiscip Rev Comput Mole Sci.

[39] Pino-Rios R, Chigo-Anota E, Shakerzadeh E, Cárdenas-Jirón G (2020). B12N12 cluster as a collector of noble gases: a quantum chemical study. Phys E Low-Dimen Syst Nanostructures.

[40] Tkatchenko A, Scheffler M (2009). Accurate molecular van der Waals interactions from ground-state electron density and free-atom reference data. Phys Rev Lett.

[41] Jessima SHM, Berisha A, Srikandan SS, Subhashini S (2020). Preparation, characterization, and evaluation of corrosion inhibition efficiency of sodium lauryl sulfate modified chitosan for mild steel in the acid pickling process. J Mol Liq.

[42] Dagdag O, Berisha A, Safi Z, Hamed O, Jodeh S, Verma C, El Harfi A (2020). DGEBA-polyaminoamide as effective anti-corrosive material for 15CDV6 steel in NaCl medium. Computational and experimental studies. J App Poly Sci.

[43] Hsissou R, Dagdag O, Abbout S, Benhiba F, Berradi M, El Bouchti M, Elharfi A (2019). Novel derivative epoxy resin TGETET as a corrosion inhibition of E24 carbon steel in 1.0 M HCl solution. Experimental and computational (DFT and MD simulations) methods. J Mol Liq.

[44] Uppalapati PK, Berisha A, Velmurugan K, Nandhakumar R, Khosla A, Liang T (2021). Salen type additives as corrosion mitigator for Ni–W alloys: detailed electronic/atomic-scale computational illustration. Int J Quan Chemis.

[45] Lu T, Chen F (2012). Multiwfn: a multifunctional wavefunction analyzer. J Comput Chemis.

[46] Humphrey W, Dalke A, Schulten K (1996). VMD: visual molecular dynamics. J Mol Graph.

[47] Rappé AK, Casewit CJ, Colwell KS, Goddard WA, Skiff WM (1992). UFF, a full periodic table force field for molecular mechanics and molecular dynamics simulations. J Am Chem Soc.

[48] El Faydy M, About H, Warad I, Kerroum Y, Berisha A, Podvorica F, Zarrouk A (2021). Insight into the corrosion inhibition of new bis-quinolin-8-ols derivatives as highly efficient inhibitors for C35E steel in 0.5 M H2SO4. J Mol Liq.

[49] Oukhrib R, Abdellaoui Y, Berisha A, Abou Oualid H, Halili J, Jusufi K, Len CDFT (2021). Monte Carlo and molecular dynamics simulations for the prediction of corrosion inhibition efficiency of novel pyrazolylnucleosides on Cu (111) surface in acidic media. Sci Rep.

[50] Berisha A (2021). Ab inito exploration of nanocars as potential corrosion inhibitors. Comput Theor Chemis.

[51] Barosi A, Berisha A, Mangeney C, Pinson J, Dhimane H, Dalko PI (2021). Efficient construction of a redox responsive thin polymer layer on glassy carbon and gold surfaces for voltage-gated delivery applications. Mater Adv.

[52] Molhi A, Hsissou R, Damej M, Berisha A, Thaçi V, Belafhaili A, El Hajjaji S (2021). Contribution to the corrosion inhibition of C38 steel in 1 M hydrochloric acid medium by a new epoxy resin PGEPPP. Int J Corros Scale Inhib.

[53] Reka AA, Pavlovski B, Fazlija E, Berisha A, Pacarizi M, Daghmehchi M, Oral A (2021). Diatomaceous earth: characterization, thermal modification, and application. Open Chemis.

[54] Khalaf B, Hamed O, Jodeh S, Bol R, Hanbali G, Safi Z, Samhan S (2021). Cellulose-based hectocycle nanopolymers: synthesis, molecular docking and adsorption of difenoconazole from aqueous medium. Int J Mol Sc.

[55] Dagdag O, Harfi A, Gana L, Safi Z, Guo L, Berisha A, El Gouri M (2021). Designing of phosphorous based highly functional dendrimeric macromolecular resin as an effective coating material for carbon steel in NaCl: computational and experimental studies. J App Poly Sci.

[56] Haldhar R, Prasad D, Bahadur I, Dagdag O, Berisha A (2021). Evaluation of Gloriosa superba seeds extract as corrosion inhibition for low carbon steel in sulfuric acidic medium: a combined experimental and computational studies. J Mol Liq.

[57] Hsissou R, Benhiba F, Echihi S, Benkhaya S, Hilali M, Berisha A, Elharfi A (2021). New epoxy composite polymers as a potential anticorrosive coatings for carbon steel in 3.5% NaCl solution: experimental and computational approaches. Chem Data Collect.

[58] Chen WH, Wu CH, Cheng HC (2011). Modified Nosé-Hoover thermostat for solid state for constant temperature molecular dynamics simulation. J Comput Phys.

[59] Rao GD, Acharya BN, Kaushik MP (2013). An efficient synthesis of β-ketoesters via transesterification and its application in Biginelli reaction under solvent-free, catalyst-free conditions. Tetra Lett.

[60] Hamed O, Lail BA, Deghles A, Qasem B, Azzaoui K, Obied AA, Jodeh S (2019). Synthesis of a cross-linked cellulose-based amine polymer and its application in wastewater purification. Environ Sci Poll Res.

[61] Gül ÜD, Şenol ZM, Gürsoy N, Şimşek S (2019). Effective UO^22+^ removal from aqueous solutions using lichen biomass as a natural and low-cost biosorbent. J Environ Radioact.

[62] Uddin MK (2017). A review on the adsorption of heavy metals by clay minerals, with special focus on the past decade. Chem Eng J.

[63] Adeyemo AA, Adeoye IO, Bello OS (2017). Adsorption of dyes using different types of clay: a review. App Wat Sci.

[64] Bo S, Ren W, Lei C, Xie Y, Cai Y, Wang S, Gao J, Ni Q, Yao J (2018). Flexible and porous cellulose aerogels/zeolitic imidazolate framework (ZIF-8) hybrids for adsorption removal of Cr(IV) from water. J Solid State Chemis.

[65] Chwastowski J, Staroń P, Kołoczek H, Banach M (2017). Adsorption of hexavalent chromium from aqueous solutions using Canadian peat and coconut fiber. J Mol Liq.

[66] Lin J, Chen X, Chen C, Hu J, Zhou C, Cai X, Wang W, Zheng C, Zhang P, Cheng J (2018). Durably antibacterial and bacterially anti-adhesive cotton fabrics coated by cationic fluorinated polymers. ACS App Mater Inter Sci.

[67] Da Silva JS, Da Rosa MP, Beck PH, Peres EC, Dotto GL, Kessler F, Grasel FS (2018). Preparation of an alternative adsorbent from Acacia Mearnsii wastes through acetosolv method and its application for dye removal. J Clean Prod.

[68] Lin JX, Zhan SL, Fang MH, Qian XQ, Yang H (2008). Adsorption of basic dye from aqueous solution onto fly ash. J Environ Manage.

[69] Chakraborty R, Asthana A, Singh AK, Jain B, Susan ABH (2020). Adsorption of heavy metal ions by various low-cost adsorbents: a review. Int J Environ.

[70] Gupta S, Babu BV (2009). Utilization of waste product (tamarind seeds) for the removal of Cr(VI) from aqueous solutions: equilibrium, kinetics, and regeneration studies. J Environ Manag.

[71] Guo L, Zhang ST, Li WP, Hu G, Li X (2014). Experimental and computational studies of two antibacterial drugs as corrosion inhibitors for mild steel in acid media. Mater Corros.

[72] Hsissou R, Benzidia B, Rehioui M, Berradi M, Berisha A, Assouag M, Elharfi A (2020). Anticorrosive property of hexafunctional epoxy polymer HGTMDAE for E24 carbon steel corrosion in 10 M HCl: gravimetric, electrochemical, surface morphology and molecular dynamic simulations. Polym Bull.

[73] Dagdag O, Hsissou R, Berisha A, Erramli H, Hamed O, Jodeh S, El Harfi A (2019). Polymeric-based epoxy cured with a polyaminoamide as an anticorrosive coating for aluminum 2024–T3 surface: experimental studies supported by computational modeling. J Bio- Tribo-Corros.

[74] Hsissou R, Dagdag O, Abbout S, Benhiba F, Berradi M, El Bouchti M, Elharfi A (2019). Novel derivative epoxy resin TGETET as a corrosion inhibition of E24 carbon steel in 1.0 M HCl solution. Experimental and computational (DFT and MD simulations) methods. J Mol Liq.

[75] Abbout S, Zouarhi M, Chebabe D, Damej M, Berisha A, Hajjaji N (2020). Galactomannan as a new bio-sourced corrosion inhibitor for iron in acidic media. Heliyon.

[76] Dagdag O, Hsissou R, El Harfi A, Berisha A, Safi Z, Verma C, El Gouri M (2020). Fabrication of polymer based epoxy resin as effective anti-corrosive coating for steel: computational modeling reinforced experimental studies. Surf Interfaces.

[77] Amrhar O, Berisha A, El Gana L, Nassali H, Elyoubi S, M.  (2021). Removal of methylene blue dye by adsorption onto Natural Muscovite Clay: experimental, theoretical and computational investigation. Int J Environ Anal Chem.

[78] Alahiane M, Oukhrib R, Berisha A, Albrimi YA, Akbour RA, Abou Oualid H, Hamdani M (2021). Electrochemical, thermodynamic and molecular dynamics studies of some benzoic acid derivatives on the corrosion inhibition of 316 stainless steel in HCl solutions. J Mol Liq.

[79] Berisha A (2021). First principles details into the grafting of aryl radicals onto the free-standing and borophene/Ag (1 1 1) surfaces. Chem Phys.

[80] Bridgeman AJ, Cavigliasso G, Ireland LR, Rothery J (2001). The Mayer bond order as a tool in inorganic chemistry. J Chem Soc Dalton Trans.

[81] Stevenson J, Sorenson B, Subramaniam VH, Raiford J, Khlyabich PP, Loo YL, Clancy P (2017). Mayer bond order as a metric of complexation effectiveness in lead halide perovskite solutions. Chem Mater.

